# Integrated models of population pharmacokinetics and exposure response to optimize dosage regimen for anaprazole sodium in duodenal ulcer

**DOI:** 10.1016/j.ejps.2024.106781

**Published:** 2024-07-01

**Authors:** Lei Zhang, Ling Song, Cheng Cui, Chunyang Wang, Yi Zhang, Xueting Yao, Dongyang Liu

**Affiliations:** a Department of Cardiology and Institute of Vascular Medicine, Peking University Third Hospital, Beijing, 100191, PR China; b Drug Clinical Trial Center, Peking University Third Hospital, Beijing, 100191, PR China; c Center of Clinical Medical Research, Institute of Medical Innovation and Research, Peking University Third Hospital, Beijing, 100191, PR China

**Keywords:** Anaprazole sodium, Duodenal ulcer, Population pharmacokinetics, Exposure-response model, Dosage regimen

## Abstract

Anaprazole sodium enteric-coated tablet is a novel proton pump inhibitor which has been approved for the treatment of duodenal ulcer. The aim of this study is to provide reliable information for the design of an optimal dosage regimen. Population pharmacokinetics and exposure-response models were integrated to evaluate the pharmacokinetic parameters and covariates of Anaprazole and its metabolite M21-1, and subsequently provided dosage suggestions based on clinical trials and simulation data. A pharmacokinetic model incorporating two-compartment for the parent drug and one-compartment for the metabolite, with both first-order and zero-order mixed absorption was used to describe the pharmacokinetics of Anaprazole and M21-1. Age emerged as a significant covariate affecting the elimination rate constant of M21-1, with clearance decreasing as age advances. No correlation was observed between the pharmacokinetics of Anaprazole or M21-1 and the adverse reactions under the current dosages. BMI might be the influence factor of the mild gastrointestinal adverse reactions. Meanwhile, Anaprazole had a good healing rate (94.0 %) in duodenal ulcer patients and the exposure-response analysis indicated that the cured results were not influenced by the exposure parameters of parent drug or metabolite. In conclusion, the drug is safe when dosing between 20 and 100 mg once a day.

## Graphical abstract

**Figure:**
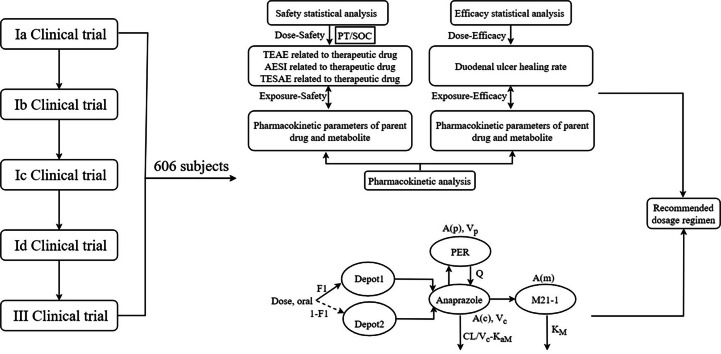

## 1 Introduction

Peptic ulcer affects approximately 10 % of the population and requires drug therapy (Lanas and Chan, 2017). In China, the in-hospital mortality rate due to peptic ulcer is 0.35 % (Zheng et al., 2020). Proton pump inhibitors (PPIs) are the first-line drugs for treating peptic ulcer and are among the best-selling medications in the world. Compared to commonly used gastric acid suppressant drugs like H2 receptor antagonists, PPIs offer great advantages in inhibiting gastric acid secretion with a completely inhibitory effect and rapid onset. Moreover, as an irreversible process, PPIs provide long-lasting inhibition and can inhibit the secretion of basic gastric acid and acid secretion caused by histamine, acetylcholine, gastrin, and food stimulation (Sachs, 1997).

Currently, there are six PPIs available on the Chinese market including lansoprazole, pantoprazole, omeprazole, esomeprazole, rabeprazole, and ilaprazole. Meanwhile, an innovative oral PPI developed by Xuanzhu Biopharm with a novel structure has been approved recently in Chinese market. Despite their satisfactory therapeutic effects, some adverse clinical effects have been reported with respect to PPIs, especially for long-term treatment, which is a common situation nowadays (Johnson and Oldfield, 2013). Recent studies have linked PPI administration to a range of unexpected effects (Targownik, 2018), which might be caused by various factors including off-target effects and CYP2C19 gene polymorphism (Gawrońska-Szklarz et al., 2012; Johnson and Oldfield, 2013). Studies have reported significant individual differences in pharmacokinetics (PK) and pharmacodynamics (PD), as well as drug interactions (Furuta et al., 2001; Shirai et al., 2001; Tanaka et al., 1997) when using some of the PPIs. However, the degree of factors influencing drug exposure or efficacy are often challenging to ascertain. Therefore, utilizing a model-based approach becomes imperative to establish the basis for designing dosage regimens for this kind of drugs, especially for new drugs without too much clinical data.

The relationship between dose/exposure and response (D-E-R) is a useful tool to inform dosage and study design for clinical studies in each stage of development. With the results, investigators are expected to better formulate the strategy for the next phase of development. In recent years, d-E-R analysis had become a widely used method in drug development (Grenier et al., 2018; Miller et al., 2005). According to some reports, the likelihood of success in confirmatory studies has been improved with the use of d-E-R analysis in various diseases. In addition, due to ethical or clinical trial design limitations, some studies can only be conducted with sparse sampling which making it impossible to obtain complete pharmacokinetic curves and corresponding parameters. Population pharmacokinetics (PPK), however, can make full use of clinical sparse sampling data for analysis to estimate individual pharmacokinetic parameters and identify physiological and pathological factors that affect pharmacokinetics. This approach has received widespread attention from regulatory authorities (FDA, 2022). According to previous studies (Lew, 1999; Shimizu et al., 2019), PPIs are eliminated rapidly in vivo, to the extent that they cannot be detected in the blood shortly after administration. This poses a challenge for obtaining PK parameters and confirming the relationship between exposure and response. Coupled with the issue of individual differences, d-E-R analysis and PPK analysis are both very effective tools for the clinical development and usage of PPIs.

Anaprazole sodium exhibits high target selectivity and undergoes metabolism via non-CYP enzymes and multienzyme pathways, making the drug safer and less prone to individual differences [24]. M21-1 is the principal metabolite of Anaprazole (accounting for 48.0–52.4 % of AUC_0–_24 h after administration of 20 or 40 mg Anaprazole), and in vitro results indicate that its inhibition of H+/K+-ATPase enzyme is comparable to that of the parent drug. Previous preclinical and clinical studies have shown that Anaprazole sodium has better safety and efficacy than most of its competitors (Shu et al., 2021; Tang et al., 2020; Zhu et al., 2022). To evaluate the covariates that affect the PK and PD of Anaprazole sodium, and recommend a suitable dosage regimen based on efficacy and safety data, a modeling approach to quantitative analyze the d-E-R relationship of Anaprazole sodium and its metabolite from multiple clinical trials was employed. As only sparse samples were obtained from the phase III clinical trial, a PPK model was also established to obtain the pharmacokinetic parameters and potential covariates influencing the PK process. The results will help to confirm the optimal typical dosage and quantitatively understand the d-E-R relationship of Anaprazole sodium in patients with duodenal ulcers, as well as determine the individualized dosing requirements for duodenal ulcer by revealing significant factors influencing the E-R relationship and quantifying their extent.

## 2 Material and methods

### 2.1 Subjects

Four phase I clinical trials (clinical trials numbers: CTR20140510, CTR20150449, CTR20150765, and CTR20190520) and one phase III clinical trial (clinical trial number: CTR20192626) were conducted to explore the safety and efficacy of Anaprazole sodium enteric-coated tablet in healthy and duodenal ulcer volunteers. The dosage and number of subjects in phase I study were listed in Table S1. In the phase III study, the dosage is 20 mg, and all subjects receive a 4-week regimen of oral administration. The PK and PD data were evaluated and used for PPK and d-E-R analysis. A total of 606 subjects were included in the study, among which 350 subjects were administrated with Anaprazole sodium enteric-coated tablet, 229 subjects with Rabeprazole sodium enteric-coated tablet and 27 subjects with placebo.

### 2.2 Software

The R software (version 3.5.3, MathSoft, Inc.) was used to organize and describe all data, conduct the Exploratory Data Analysis (EDA), and plot in the PPK study. NONMEM (version 7.2, ICON plc.) was used for model analysis, and PIRANA (version 2.8.0, Pirana Software Solutions Pty Ltd.) was used to call NONMEM programs for analysis in the PPK study. The E-R data processing was conducted using SAS software (version 9.4, SAS Institute Inc.).

### 2.3 Population pharmacokinetics model development

209 subjects with PK information were included in the final analysis dataset for the PPK model, of which 126 were healthy subjects and 83 were duodenal ulcer patients. A stepwise approach was used to model and evaluate the PK concentrations of Anaprazole sodium and M21-1. The model for the parent drug was confirmed firstly by comparing the fitting results of different structural models assuming one- and two-compartment disposition with different absorption models, such as zero- and first-order absorption (with or without a lag time). Once the PK parameters of Anaprazole for all individual subjects were obtained, a metabolite model driven by the concentration of the parent drug was established. In the model development process, the first-order conditional estimation with interaction (FOCEI) method was used for model evaluation. Interindividual variation (IIV) of PK parameters was described using an exponential model.

Several covariates were evaluated to determine whether they could influence the pharmacokinetics of Anaprazole sodium and M21-1. These covariates included age, height, weight, BMI, body surface area, gender, alanine aminotransferase (ALT), aspartate aminotransferase, total protein, total bilirubin, direct bilirubin, alkaline phosphatase, creatinine, urine protein, ketones, white blood cell count, neutrophil percentage, lymphocyte percentage, red blood cell count, hemoglobin, platelet count, creatine kinase, activated partial thromboplastin time, prothrombin time, whether the patient has duodenal ulcer or not, CYP3A4 gene polymorphism rs2242480 (g.99763843C>T), CYP3A5 gene polymorphism rs776746 (g.99672916T>C), CYP2C19 gene polymorphisms rs12769205 (g.94775367A>G), rs3758581 (c.991A>G) and rs4986893 (c.636G>A), and gene phenotype. A stepwise approach was utilized to incorporate covariates into the model for covariate screening. Firstly, forward selection was employed to include covariates (α<0.01, with a change in the minimum objective function value (MOFV) >6.63 when df=1), gradually incorporating eligible covariates until the full model was generated. Subsequently, backward elimination was used to sequentially remove covariates (α<0.001, with a change in MOFV <10.83 when df=1), until only covariates with significant impact (*p* < 0.001) remained in the model, thus forming the final covariate model.

Goodness-of-fit (GOF) plots were used to evaluate the deviation of the final model fit. Visual predictive checks (VPC) were used to evaluate the model. The final model was simulated 1000 times using NONMEM software and the results were compared with the measured results based on the 10th, 50th (median) and 90th percentile distribution-time plots of the simulated predicted concentration. The measured data and/or percentile distribution based on the measured data were overlaid to create a VPC plot to evaluate the consistency between the model-based data and the measured data. The original data set was also resampled 500 times (Bootstrap) to observe whether the final model parameters fell within the 95 % confidence interval (CI) of the Bootstrap parameter results.

Based on the final population model and clinical trial requirements, the plasma drug concentration-time curve was simulated and plotted for the clinical trial dosing regimen.

### 2.4 Exploratory dose-exposure-response evaluation for safety and healing rate endpoint

For safety data, the incidence rate of drug-related Treatment Emerged Adverse Events (TEAEs) was very low. A total of 52 subjects reported 81 TEAEs. The majority of TEAEs were assessed as mild, with 8 TEAEs considered moderate. Statistical analysis was conducted on TEAEs, and those with an incidence rate >1 % were selected for d-E-R analysis. These included flatulence, elevated ALT, diarrhea, and abdominal distention (PT classification), as well as gastrointestinal diseases and various examinations (SOC classification).

For efficacy data, the healing rate (endpoint criteria, in duodenal ulcer patients) was considered as the efficacy parameters in this study. Finally, 83 out of 222 subjects who had sparse pharmacokinetic data from the phase III clinical trial was included in the study for the establishment of models.

The preliminary research results indicate that M21-1 is the primary metabolite of Anaprazole and exhibits comparable activity to Anaprazole. Therefore, the dosages of parent drug, along with the pharmacokinetic parameters (C_max_, AUC_0-t_, AUC_ss_, and C_ss_min_) of both Anaprazole sodium and M21-1 were used for the d-E-R analysis.

An analysis dataset was generated and any missing data points were excluded from the analysis without imputation. The safety and efficacy indicators were statistically described by dose group and the dose-response relationship was established.

To explore the potential associations between dose/exposure and response outcomes, the dose/exposure parameters were grouped by quartiles or binary. Stacked and box plots were used to visualize the relationships between dose/exposure and response.

As EDA revealed a potential correlation between some exposure parameters and safety indicators (TEAE occurred or not). To further investigate this relationship, a logistic regression model was employed to analyze the association between exposure parameters and response indicators. Specifically, single-factor Logistic regression analysis was employed initially to examine various safety indicators and exposure parameters. Furthermore, study design types (single-dose/multiple-dose), subject types (healthy individuals/patients), age, gender, BMI, and the AUC_ss_ of the metabolite M21-1 were included for multivariate Logistic analysis. Stepwise selection was utilized with a significance probability (p value) of 0.15 for including variables and 0.05 for excluding variables.

## 3 Results

### 3.1 Preliminary data analysis

**Fig. 1:**
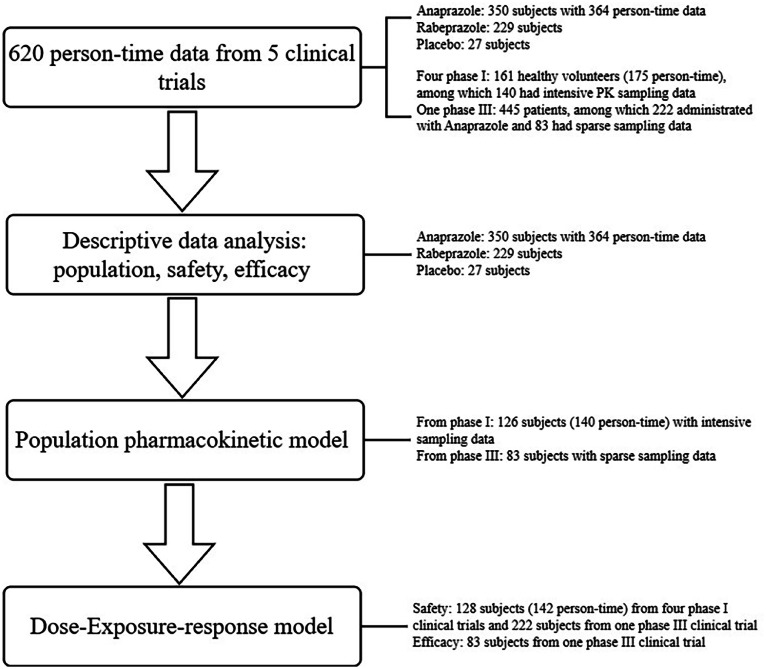
Description of the involved populations in each analysis.

The data of this study was collected from 5 clinical trials including 606 subjects. A two-cycle crossover trial was designed with 14 subjects in food effect study and a final of 620 person-time data was collected. To provide a clear description of the involved populations in each analysis, a flowchart was created as shown in Fig. 1. The details of the data included in the study was shown in Table 1. The 364 person-time data who received Anaprazole sodium enteric-coated tablets in each study and each dose group was shown in Table S1.

**Table 1:** Demographic summary of five clinical trials.

	Whole population (Cases, *N* = 620*)	phase I population (without positive control, Cases, *N* = 169*)	Population pharmacokinetics analysis population (Cases, *N* = 223*)	Safety analysis population (Cases, *N* = 364*)	Efficacy analysis population included in E-R analysis (healing rate of duodenal ulcer, *N* = 83)
Age (year) Mean (SD) Median (CV%) (Min, Max) BMI (kg/m^2^) Mean (SD) Median (CV%) (Min, Max) Gender Male Clinical trial No. CTR20140510 CTR20150449 CTR20150765 CTR20190520 CTR20192626	38.77±11.41 37.00 (29.44 %) (18.00–69.00) 22.67±2.83 22.70 (12.50 %) (12.90–35.70) 382 (61.61 %) 66 (10.65 %) 28 (4.52 %) 36 (5.81 %) 45 (7.26 %) 445 (71.77 %)	30.54±6.50 30.00 (21.28 %) (18.00–45.00) 22.44±1.60 22.50 (7.15 %) (19.10–26.10) 100 (59.17 %) 66 (39.05 %) 28 (16.57 %) 36 (21.30 %) 39 (23.08 %) NA	35.12±10.07 33.00 (30.51 %) (18.00–69.00) 22.74±2.30 22.70 (10.14 %) (18.00–30.90) 120 (57.42 %) 52 (23.32 %) 28 (12.56 %) 30 (14.35 %) 30 (13.45 %) 83 (37.22 %)	36.88±10.83 35.00 (29.37 %) (18.00–69.00) 22.62±2.66 22.60 (11.74 %) (12.90–30.90) 226 (62.09 %) 54 (14.84 %) 28 (7.69 %) 30 (8.24 %) 30 (8.24 %) 222 (60.99 %)	42.18±10.56 43.00 (25.03%) (24.00–69.00) 23.17±3.05 23.10 (13.15%) (18.00–30.90) 50 (60.24%) NA NA NA NA 83 (100.00 %)

*: A total of 14 subjects participated in the food effect (CTR20150449) study, 620 person-time data was included. The others were similar.

For subjects who used Anaprazole sodium enteric-coated tablets, 140 cases had intensive PK sampling data. The C_max_, C_min_ and AUC_0-t_ after single administration and at steady-state were calculated according to the measured values. Among which, a total of 60 subjects who received multiple doses of Anaprazole sodium (clinical trial No. CTR20150765 and CTR20190520) had the steady C_max,ss_, C_min,ss_, and AUC_ss_ data of parent drug and the metabolite M21-1. In CTR20192626 study, 83 out of 222 subjects had sparse sampling data and their pharmacokinetic parameters containing C_max_, C_min_, and AUC_0-t_ were simulated with a PPK model.

**Fig. 2:**
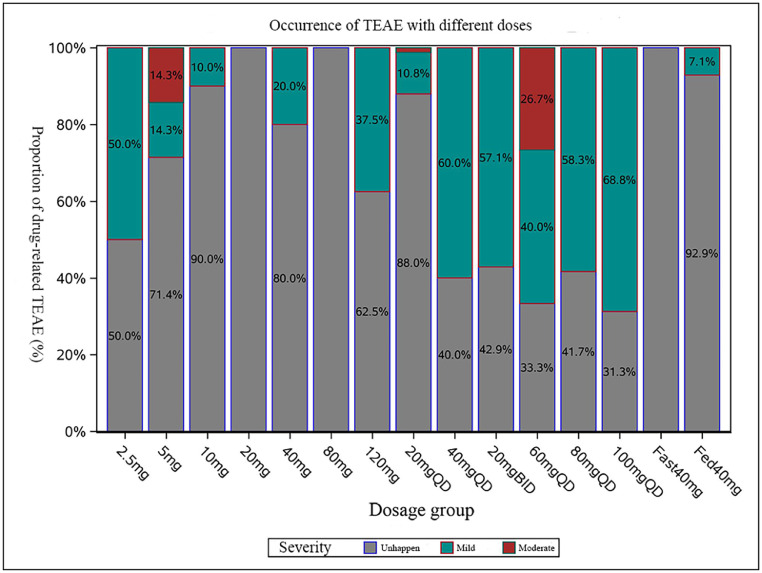
TEAE related with therapy drug in different dose groups among the total population.

No severe AEs occurred in the five clinical trials. For 364 volunteers who received Anaprazole sodium enteric-coated tablets, the TEAE related with therapy drug in different dose groups among the total population was described and plotted as Fig. 2. The incidence of drug-related AEs was 14.3 % in subjects receiving Anaprazole sodium enteric-coated tablets and the data for Rabeprazole was 12.4 %. In addition, the incidence of AEs was 33.3 % in the placebo group.

When using the PT classification, four kinds of AEs were monitored with occurrence rate more than 1 %, including flatulence (2.47 %), increased ALT (1.92 %), diarrhea (1.10 %), and abdominal distension (1.10 %). When using SOC as classification criterion, a total of 24 subjects were reported with gastrointestinal diseases (6.59 %) and 22 subjects were reported with variety of examinations (6.04 %). In addition, a total of 6 subjects (1.65 %) were reported drug-related AEs of special concern (AESI, 1.65 %), including 2 (0.55 %) elevated TSH, 3 (0.82 %) abnormal liver function and 1 (0.27 %) prolonged QT interval.

### 3.2 Evaluation of population pharmacokinetics model and covariates

**Fig. 3:**
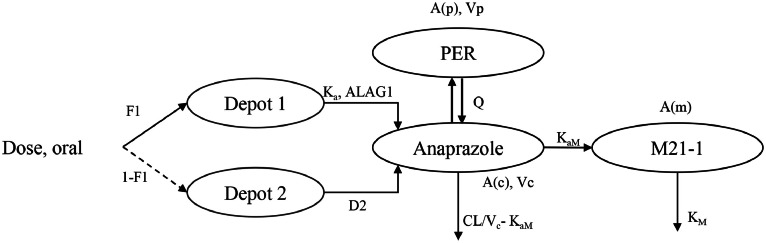
Final population pharmacokinetics model structure of Anaprazole sodium and its metabolite M21-1. F1 is the Fraction of drug absorbed in the first compartment, D2 is the duration of zero-order infusion, K_a_ is the first-order absorption rate constant, ALAG1 is the absorption lag for first-order absorption, V_c_ is the volume of parent drug distribution in central compartment, V_p_ is the volume of parent drug distribution in peripheral compartment, CL is the systemic clearance of parent drug, Q is the distribution clearance of parent drug, K_aM_ is the formation rate constant of metabolite M21-1 from parent drug, K_M_ is the elimination rate constant of metabolite M21-1, A(c) is the mass of parent drug in central compartment, A(p) is the mass of parent drug in peripheral compartment and A(m) is the concentration of metabolite M21-1.

A model with a mixed absorption of first and zero order, including absorption delay, two-compartment model for parent drug, one-compartment model for metabolite was able to describe the PK characteristics of Anaprazole sodium and M21-1. The specific formulas were as follows and the final model structure was shown in Fig. 3.

(1) $$\frac{\text{dA}\left(1\right)}{\text{dt}}=-K_{a}*A\left(1\right)$$

(2) $$\frac{\text{dA}\left(c\right)}{\text{dt}}=K_{a}*A\left(1\right)-\left(\frac{\text{CL}}{V_{c}}-K_{\text{aM}}\right)*A\left(c\right)-K_{\text{aM}}*A\left(c\right)+\frac{Q}{V_{p}}*A\left(p\right)-\frac{Q}{V_{c}}*A(c)A(c)\left(0\right)=0\;$$

(3) $$\frac{\text{dA}\left(p\right)}{\text{dt}}=-\frac{Q}{V_{p}}*A\left(p\right)+\frac{Q}{V_{c}}*A\left(c\right)A(p)\left(0\right)=0$$

$$C_{c}=\frac{A\left(c\right)}{V_{c}}$$

(4) $$\frac{\text{dA}\left(m\right)}{\text{dt}}=K_{\text{aM}}*C_{c}-K_{M}*A\left(m\right)\;A(m)\left(0\right)=0$$

Where V_c_ is the volume of parent drug distribution in central compartment, V_p_ is the volume of parent drug distribution in peripheral compartment, CL is the systemic clearance of parent drug, Q is the distribution clearance of parent drug, K_a_ is the first-order absorption rate constant of parent drug, K_aM_ is the formation rate constant of metabolite M21-1 from parent drug, K_M_ is the elimination rate constant of metabolite M21-1, C_c_ is the concentration of parent drug in central compartment, A(1) is the mass of parent drug in first-order absorption compartment, A(c) is the mass of parent drug in central compartment, A(p) is the mass of parent drug in peripheral compartment, and A(m) is the concentration of metabolite M21-1.

A proportional error model was used to describe the PK residual and the equations were as follows:

(5) $$P_{ij}=\theta_{i}\times exp\left(\eta_{ij}\right)$$

(6) $$Y_{obs}=Y_{pred}\times \left(1+\varepsilon 1\right)$$

P_ij_ is the value of parameter i of subject j, θ_i_ is the typical value of population parameter i, η_ij_ is the inter-individual and intra-individual variation of the parameter i of subject j which following the (0, ω^2^) normal distribution, Y_obs_ and Y_pred_ are the actual concentration and predict concentration, ε1 is the residual which following the (0, ***σ***^2^) normal distribution.

The covariates analysis was conducted after the selection of the base model. The descriptive statistics for continuous covariates were presented in Table S2 and for categorical covariates were presented in Table S3. Using the basic model, the correlation coefficient matrix was calculated through correlation analysis and significantly correlated variables to the PK parameters of parent drug and M21-1 (Pearson correlation coefficient test *p* < 0.05) were selected as potential covariates for investigation.

Among the covariates, it was found that age had a significant impact on the elimination rate constant (K_M_) of the metabolite M21-1. The inclusion of age (AGE) resulted in a decrease of 11.983 in the OFV of the model. The quantitative relation between AGE and K_M_ of the metabolite M21-1 was shown as follow:

(7) $$K_{M}=\theta_{KM\;}\times \;{\left(\frac{\text{AGE}}{39}\right)}^{-0.365}\times e^{\eta K_{M}}$$

Where K_M_ is the elimination rate constant of the metabolite M21-1, θ_KM_ is the typical value of K_M_, η_KM_ is the random effect of K_M_.

**Fig. 4:**
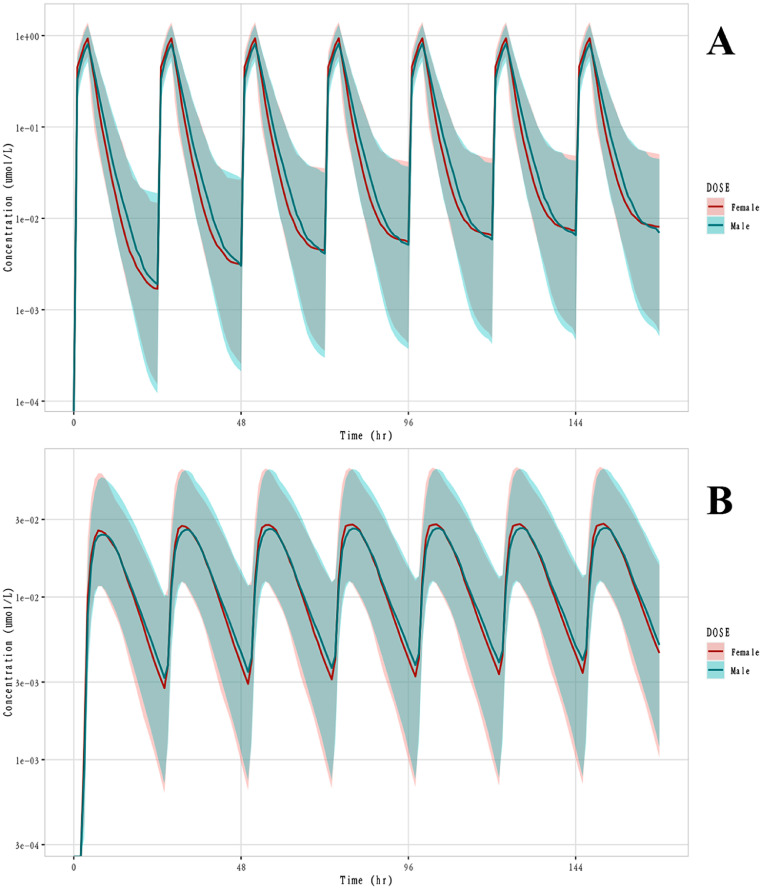
Simulated concentration-time curve in different genders. Colored solid line: the median of the simulated drug concentration; Colored area: the 90 % confidence interval of the median of the simulated drug concentration; A: parent drug; B: metabolite M21-1.

In addition, gender was found moderately correlated with the central volume of parent drug distribution (V_c_). To explore the degree and reasons for the influence of gender, variance analysis was used to investigate the effect of gender on the measured PK parameters (obtained through NCA). The results showed that only the 60 mg dose group showed a significant effect of gender on AUC and C_max_. To further explore the effect of gender on the final exposure, the exposure of the parent drug and metabolites in different sexes was simulated. The plasma concentration-time curves of male and female subjects were simulated for the 20 mg once-daily dosing regimen (phase III clinical dosing regimen). The results (Fig. 4) showed that the exposure of Anaprazole and M21-1 in females and males was similar with a 90 % CI overlap. Therefore, gender was not included as a significant covariate in the final model.

**Fig. 5:**
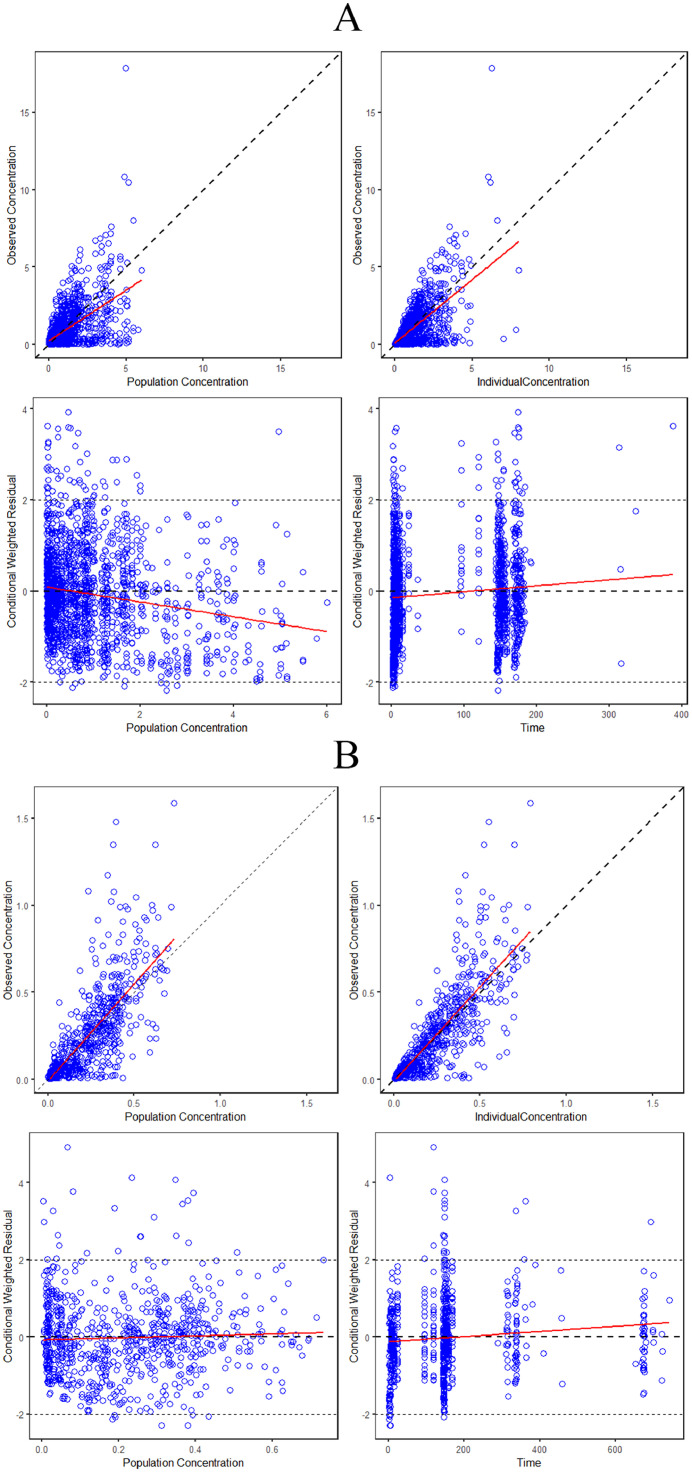
GOF plots of the final model for the parent drug and metabolite M21-1. A: parent drug; B: metabolite M21-1.

The final estimated PK parameters of the model were presented in Table 2, while the GOF plots of the final model for the parent drug and M21-1 were depicted in Fig. 5. From the result, there was a slight underestimation phenomenon for the parent drug at high concentrations. However, the overall fit remained within an acceptable range.

**Table 2:** Estimates of population pharmacokinetics model parameters of Anaprazole sodium and its metabolite M21-1.

Parameter	Unit	Theta		Omega		Shrinkage (%)	Bootstrap	
		Estimate	RSE (%)	IIV (%)	RSE (%)		Estimate	(95 %CI)
F1	NA	0.250	23.5	0 (Fixed)	NA	100	0.262	(0.115, 0.424)
D2	Hr	4.23	1.80	0 (Fixed)	NA	100	4.24	(4.06, 4.43)
K_a_	hr^−1^	0.457	12.2	0 (Fixed)	NA	100	0.462	(0.370, 0.521)
CL	L/hr	10.2	4.60	30.8	12.6	10.3	10.1	(9.09, 11.2)
V_c_	L	16.9	9.40	54.3	23.6	16.5	16.9	(13.3, 20.7)
Q	L/hr	0.773	25.6	88.7	36.2	40.6	0.775	(0.285, 1.77)
V_p_	L	133	60.9	0 (Fixed)	NA	100	135	(12.8, 677)
ALAG1	hr	2.64	6.30	0 (Fixed)	NA	100	2.66	(2.25, 2.94)
K_aM_	hr^−1^	0.0486	8.10	34.2	15.5	36.3	0.0487	(0.0422, 0.0582)
K_M_	hr^−1^	0.143	4.40	22.1	14.0	21.8	0.144	(0.132, 0.157)
AGE on K_M_	NA	−0.365	24.8	NA	NA	NA	−0.362	(−0.540, −0.176)
Epsilon_Ana	NA	0.325	5.50	NA	NA	4.20	0.323	(0.290, 0.357)
Epsilon_21–1	NA	0.246	7.30	NA	NA	4.90	0.248	(0.206, 0.287)

NA: Not applicable; IIV: Inter-individual variability; F1: Fraction of drug absorbed in the first compartment; D2: Duration of zero-order infusion; K_a_: First-order absorption rate constant; CL: Systemic clearance of the parent drug; V_c_: Distribution volume of the central compartment of the parent drug; Q: Inter-compartmental clearance of the parent drug; V_p_: Distribution volume of the peripheral compartment of the parent drug; K_aM_: Rate constant for the generation of metabolites driven by the parent drug; K_M_: Elimination rate constant of metabolites; ALAG1: Absorption lag for first-order absorption; Epsilon_Ana: Proportional residual error for the parent drug; Epsilon_21–1: Proportional residual error for the metabolite.

Internal validation was performed using the bootstrap method and the success rates of the parent drug and metabolite models were 82.4 % and 100.0 %, respectively. The median values of the 500 bootstrap results were in close agreement with the final model parameter estimates and the 95 % confidence intervals encompassed the final model parameter estimates, indicating that the final model was robust and the parameter estimates were accurate. The bootstrap results were detailed in Table 2.

**Fig. 6:**
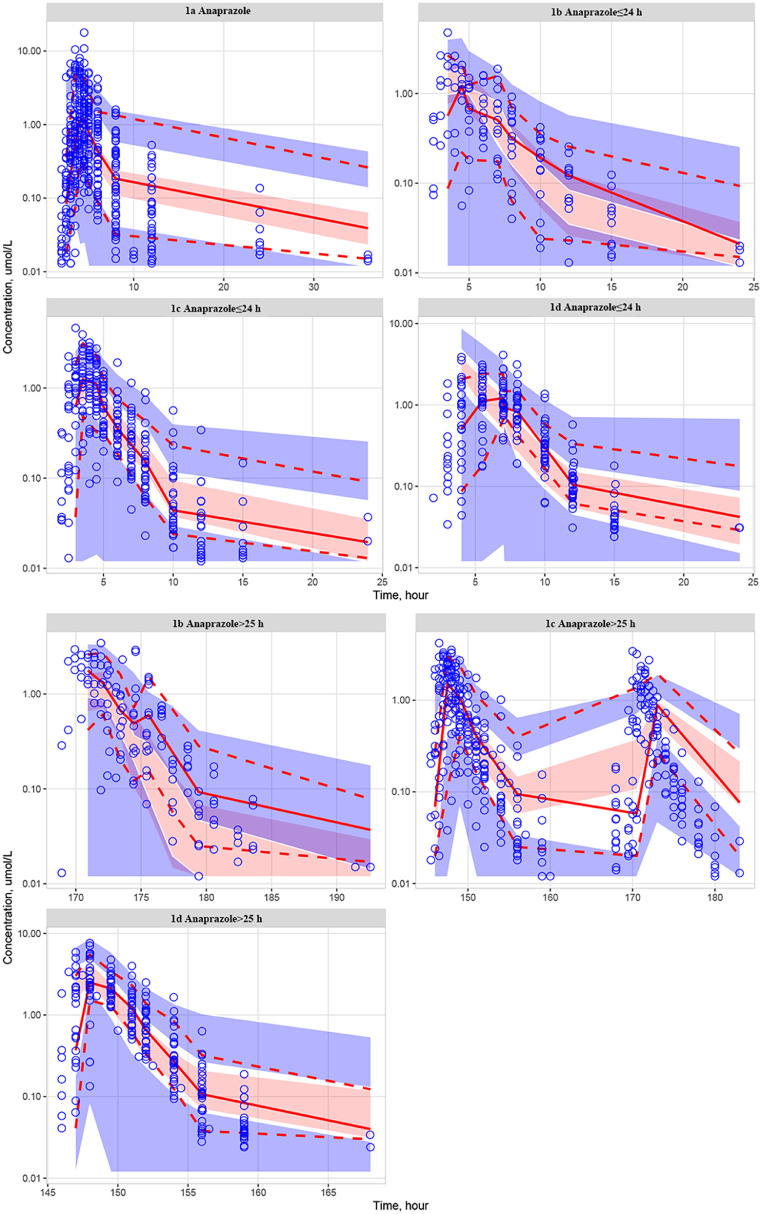
The VPC results for the parent drug. Blue hollow circles: the observed values; Red solid and dashed lines: 50 %, 10 %, and 90 % quantiles of the observed values; Red shaded area: 95 % confidence interval of the 50 % quantile of the simulated values; Blue shaded area: 95 % confidence interval of the 10 % and 90 % quantiles of the simulated values; 1a corresponds to the clinical trial with the No. CTR20140510; 1b corresponds to the clinical trial with the No. CTR20150449; 1c corresponds to the clinical trial with the No. CTR20150765; 1d corresponds to the clinical trial with the No. CTR20190520.

**Fig. 7:**
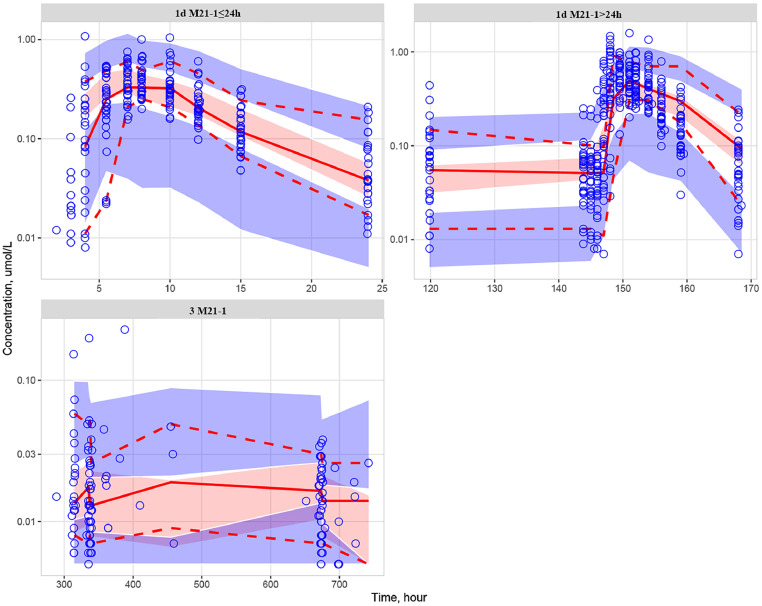
The VPC results for the metabolite M21-1. Blue hollow circles: the observed values; Red solid and dashed lines: 50 %, 10 %, and 90 % quantiles of the observed values; Red shaded area: 95 % confidence interval of the 50 % quantile of the simulated values; Blue shaded area: 95 % confidence interval of the 10 % and 90 % quantiles of the simulated values; 1d corresponds to the clinical trial with the No. CTR20190520; 3 corresponds to the clinical trial with the No. CTR20192626.

The model was also evaluated using VPC by simulating the model 1000 times using NONMEM 7.2 software. The VPC results for the parent drug and M21-1 were presented in Fig. 6, Fig. 7, respectively. The plots indicated that most of the concentration estimates were within an acceptable range, although some slight bias was observed.

**Fig. 8:**
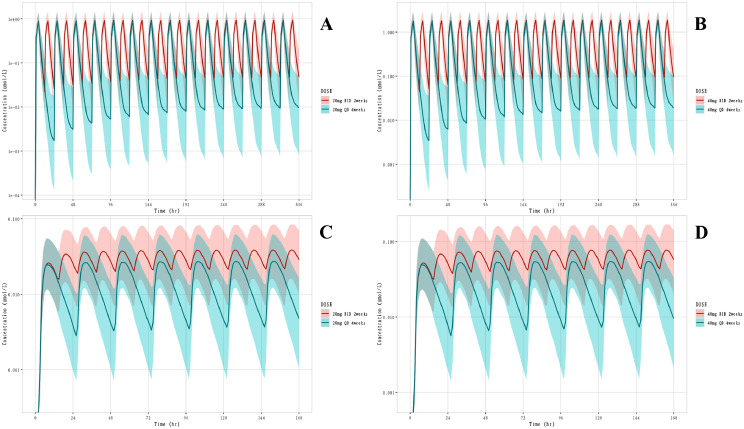
Simulation concentration-time curve of Anaprazole and M21-1. A: Concentration-time curve of Anaprazole with 20 mg BID (2 weeks) or QD (4 weeks) administration; B: Concentration-time curve of Anaprazole with 40 mg BID (2 weeks) or QD (4 weeks) administration; C: Concentration-time curve of M21-1 with 20 mg BID (2 weeks) or QD (4 weeks) administration; D: Concentration-time curve of M21-1 with 40 mg BID (2 weeks) or QD (4 weeks) administration.

Simulation was conducted using NONMEM 7.2 and four dosing regimens were simulated based on the final PPK model. The simulation results for Anaprazole and M21-1 were shown in Fig. 8. The plots indicated that for the same dose, the difference in C_max_ of Anaprazole between twice-daily and once-daily dosing was small, while the C_max_ of M21-1 was higher in twice-daily dosing.

### 3.3 Exposure-efficacy analysis results in duodenal ulcer population

The healing rate of 83 subjects with sparse sample data was 94.0 % (78/83), of which 17 were cured (20.5 %), 61 were marked effective (73.5 %), 2 were effective (2.41 %) and 3 were ineffective (3.61 %). The results were comparable to the duodenal ulcer healing rate evaluated in all 215 subjects (222 patients received Anaprazole sodium in phase III clinical trial and 215 of them had healing rate data) (Table 3). A binary sort as recovery and non-recovery based on the diagnosis of duodenal ulcer was used. Further single logistic regression analysis was conducted to screen the influence factors to the healing of duodenal ulcer. The results were shown as Table 4. Accordingly, the cured results were not influenced by the exposure parameters of parent drug or metabolite M21-1.

**Table 3:** Evaluation of duodenal ulcer healing rate with Anaprazole sodium.

Dosage	In total	Healed		Unhealed		
Anaprazole (20 mg QD)		Cured	Marked effective	Effective	Ineffective	Unevaluable
	215	39 (18.14 %)	161 (74.88 %)	10 (4.65 %)	4 (1.86 %)	1 (0.47 %)
	83 (included in E-R analysis)	17 (20.48 %)	61 (65.44 %)	2 (2.41 %)	3 (3.61 %)	0 (0 %)

**Table 4:** Single factor analysis (logistic regression) of influencing factors to duodenal ulcer healing rate.

Dependent variable	Independent variable	P value	Odds Ratio (95 % CI)
Effectiveness (healed/unhealed)	Parent AUC_ss_	0.1675	0.999 (0.998, 1.000)
	Parent C_max_	0.3575	0.997 (0.992, 1.003)
	Parent AUC_0-t_	0.1301	0.999 (0.998, 1.000)
	Parent C_ss_min_	0.7043	0.984 (0.905, 1.070)
	Metabolite M21-1 C_ss_min_	0.2624	0.945 (0.856, 1.043)
	Metabolite M21-1 C_max_	0.0889	0.972 (0.941, 1.004)
	MetaboliteM21-1 AUC_ss_	0.1214	0.998 (0.996, 1.000)
	Metabolite M21-1 AUC_0-t_	0.1054	0.998 (0.995, 1.000)

### 3.4 Dose-exposure-safety analysis results of Anaprazole sodium and metabolite M21-1

The PK parameters C_max_, AUC_0-t_, AUC_ss_, and C_ss_min_ of the parent drug and the metabolite M21-1 were divided into groups according to quartile or binary principle and the value ranges were shown in Table S4. The distribution of drug-related TEAE, namely flatulence, elevated ALT, diarrhea and abdominal distention (PT classification), and gastrointestinal diseases and various examinations (SOC classification) in each quartile or binary group of exposure parameters were analyzed and the results were shown in Table 5.

**Table 5:** The distribution of TEAE in Anaprazole sodium and its metabolite M21-1.

Compound	PK parameter	Case No.	Grouped	Distribution of TEAE					
				flatulence	elevated ALT	diarrhea	abdominal distention	gastrointestinal diseases	various examinations
Anaprazole	C_max_	223	quartile	1,2,3,4	3	1,3	2,3,4	1,2,3,4	1,2,3,4
M21-1	C_max_	113	quartile		4	1,4	3		
Anaprazole	AUC_0-t_	223	quartile	1,2,3,4	1,2,4	2,4	1,2,3	1,2,3,4	1,2,3,4
M21-1	AUC_0-t_	113	quartile		4	2,4	3	1,2,3,4	
Anaprazole	C_ss_min_	114	binary	2	1,2	1,2	1,2	1,2	
M21-1	C_ss_min_	113	quartile		4	3	1,2	1,2,3,4	
Anaprazole	AUC_ss_	143	quartile	1,2,3,4	2,3,4	3	2,3,4	1,2,3,4	1,2,3,4
M21-1	AUC_ss_	113	quartile		4	2,3	3,4	1,2,3,4	1,2,3,4

Exploratory analysis suggested that with the increase of the C_max_ and AUC_ss_ of the parent drug and the AUC_0-t_ and AUC_ss_ of the metabolite M21-1, the occurrence of mild gastrointestinal system diseases had an increasing trend (Fig. S1). Meanwhile, with the increase of the AUC_ss_ of parent drug and the AUC_0-t_ and AUC_ss_ of the metabolite M21-1, the occurrence of various examinations had an increasing trend (Fig. S2). For further clarification, the single logistic regression analysis was adopted to screen the influencing factors of exposure parameters to mild gastrointestinal system diseases and various examinations. The results (Table S5) suggested that the AUC_ss_ of the metabolite M21-1 might be the major influencing factor to mild gastrointestinal system diseases. To confirm the influence of M21-1 AUC_ss_ and to further screen the independent variables, a multivariate logistic analysis was conducted. Finally, the result showed that AUC_ss_ of the metabolite M21-1 was not an influencing factor for the occurrence of mild gastrointestinal system diseases. BMI and subject type (healthy volunteer/patient) had a certain impact on the occurrence of mild gastrointestinal diseases. The results were shown in Table 6.

**Table 6:** Multiple-factor analysis (logistic regression) of influencing factors to mild gastrointestinal system diseases and various examinations.

Dependent variable	Independent variable	P value	Odds ratio (95 % CI)
Mild gastrointestinal system diseases (occurred/not occurred)	BMI	0.0483	1.387 (1.002, 1.919)
	Types of volunteers (health/patient)	0.0021	0.049 (0.007, 0.336)
various examinations (occurred/not occurred)	Types of volunteers (health/patient)	0.0011	0.068 (0.013, 0.343)

In the same way, AUC_0-t_ of the metabolite M21-1 and AUC_ss_ of the parent drug was believed to have the probability to influence the occurrence of various examinations, which was shown in Table S6. A multivariate logistic analysis was conducted and the results showed that only the type of subjects (healthy volunteer/patients) had a certain influence on the occurrence of various examinations. Details were also shown in Table 6.

## 4 Discussion

Previous evidence suggests that nearly 25 % of the population uses PPIs, with over 60 % of PPI users taking high doses (Shanika et al., 2023). Additionally, approximately 25 % of individuals continue using PPIs for more than one year (Hálfdánarson et al., 2018; Othman et al., 2016; report, 2019). Increasing evidence indicates a potential association between long-term PPI use and adverse reactions (Radu Tutuian, 2000), leading to higher demands for the safety of PPIs. Considering this, National Institute for Health and Care Excellence guidelines recommend reviewing PPI prescriptions at least annually and discontinuing or stopping any unnecessary medications if possible (report, 2019). Meanwhile, finding new drugs more effective and safer is also one of the important directions. In view of this present situation, guiding the clinical development and usage of PPIs based on the d-E-R relationship seems crucial to efficiently avoid excessive and unnecessary long-term use of the medication.

Due to limitations in sensitivity of detection, only 83 subjects had plasma concentration data of Anaprazole and M21-1 in the phase III clinical trial. Therefore, only this subset of the population was included in the correlation analysis of E-R. The remaining subjects without measurable PK concentration data were not included in the E-R model fitting. Furthermore, as only sparse PK concentration data was included in this population, a PPK model was further applied to simulate individual pharmacokinetic parameters. To mitigate potential biases due to the subset of subjects, we compared demographic parameters of the 83 subjects with those of the overall population. Particularly, we contrasted the healing rates of the 83 subjects with those of 215 subjects from phase III trials with duodenal ulcer healing rate data. As depicted in Table 1, Table 3, these data were comparable, thus utilizing the data from these 83 subjects for E-R analysis seems appropriate and can representative of the overall population characteristics.

After oral administration of Anaprazole enteric-coated tablets in healthy volunteers, the drug was primarily eliminated from the body in the form of metabolites. The metabolism of Anaprazole exhibited non-CYP enzyme and multi-enzyme characteristics, with M21-1 being the main active metabolite. Based on comparison, M21-1 had a similar action on H^+^/K^+^-ATPase enzyme as Anaprazole sodium, while most of the other metabolites did not show significant activity. Therefore, during the model building process, both the parent drug and M21-1 were selected for analysis.

The establishment of the PPK model was done using individuals from phase I and phase III studies who had plasma drug concentration data. A power model was used firstly and the results demonstrated that the C_max_ and AUC_0-last_ of Anaprazole were nonlinear between doses of 5 mg to 120 mg. This nonlinearity was not very evident in AUC_0-last_, but the estimate for C_max_ tended to deviate more from the criterion. From the NCA results obtained at different doses, it appears that the terminal elimination rate constant remains essentially consistent, indicating that the non-linearity in C_max_ is more likely attributable to the absorption process. Through model exploration and comparison, a two-compartment model with parallel mixed absorption and absorption delay exhibited a smaller OFV, and this model was selected as the base model for further analysis. However, based on the model estimation results (Table 2), the RSE value for the peripheral compartment of the parent drug is relatively high, indicating limited estimation for the second compartment of Anaprazole. This may be related to the current sample size, and there might be improvement with the inclusion of more clinical data in subsequent phases. In the GOF plot, there was a slight underestimation phenomenon for the parent drug at high concentrations, and with increasing time, there was a slight deviation in CWRES, possibly due to fewer sampling points over a longer duration. Additionally, in the VPC plot, it was observed that the actual median values for some results were not within the simulated intervals, and this may be derived from sample size and the combination of multiple dose groups data. Therefore, subsequent validation and confirmation of the model could be further pursued by expanding the sample size.

When establishing the metabolite model, it was difficult to determine the fraction of the parent drug converted into metabolites (FTA). Therefore, the parent drug was firstly modeled to obtain PK parameters for all individual subjects, and these PK parameters were further used to drive the metabolite model. In the initial model exploration, based on the structural analysis of various metabolites in blood, urine, and feces, as well as an analysis of the main metabolic pathways using preclinical mass balance studies, an approximate value for Anaprazole FTA was finally calculated. However, the fitting results still could not predict the pharmacokinetic processes of M21-1 accurately. Therefore, K_aM_ was introduced instead of fixed FTA to describe the rate constant for the generation of metabolite M21-1 driven by the parent drug. Through comparison, this model was able to better describe the pharmacokinetic processes of M21-1.

After thorough analysis, it could be preliminarily concluded that the exposure parameters of Anaprazole sodium and its metabolite M21-1 did not significantly influence the healing rate of duodenal ulcers, as well as safety concerns associated with the administration of the phase III dose. However, further real-world data is still needed to support this conclusion due to the inability to detect plasma drug concentrations in some of the individuals.

## 5 Conclusions

A population pharmacokinetic model was developed incorporating first-order and zero-order mixed absorption with absorption delay, which could adequately characterize Anaprazole sodium and M21-1 concentrations. Age was screened as a significant covariate to influence the elimination rate constant of M21-1.

Based on the d-E-R analysis, Anaprazole sodium demonstrated safety and tolerability within the range of single administration (2.5 mg-120 mg) or multiple administration (20 mg-100 mg QD). The results revealed no correlation between the incidence and severity of drug-related TEAE and the dose or exposure of Anaprazole sodium and M21-1. In the study involving patients with duodenal ulcer, the healing rate was 93.0 % when administered with 20 mg of Anaprazole sodium, which was comparable to the healing rate of 10 mg Rabeprazole (95.9 %). No sensitive exposure parameters affecting the healing rate were identified.

According to these findings, it can be concluded that Anaprazole sodium is safe and well-tolerated when administered at a dose of 20 mg QD or no more than 5 times the daily dose (100 mg QD), which could be referred during the clinical application.

## CRediT authorship contribution statement

**Lei Zhang:** Writing – original draft, Visualization, Validation, Investigation, Formal analysis. **Ling Song:** . **Cheng Cui:** Supervision, Methodology, Investigation, Formal analysis, Conceptualization. **Chunyang Wang:** Validation, Project administration, Formal analysis. **Yi Zhang:** Methodology, Investigation, Formal analysis. **Xueting Yao:** Writing – review & editing, Supervision, Project administration, Conceptualization. **Dongyang Liu:** Writing – review & editing, Validation, Supervision, Methodology, Funding acquisition, Conceptualization.

## Data Availability

Data will be made available on request.

## Appendix Supplementary materials

Image, application 1
