# Supplementary material for: Integrated models of population pharmacokinetics and exposure response to optimize dosage regimen for anaprazole sodium in duodenal ulcer
Source: Eur J Pharm Sci. 2024 Jul 1;198:106781. doi: 10.1016/j.ejps.2024.106781 (PMC11156759; doi:10.1016/j.ejps.2024.106781)
Supplement: Supplementary file 1 [file mmc1.docx]

Table S1 Distribution of 364 person-time data who received Anaprazole sodium enteric-coated tablets in each study and each dose group

| Dosage | CTR20140510 | CTR20150449 | CTR20150765 | CTR20190520 | CTR20192626 | Total |
| --- | --- | --- | --- | --- | --- | --- |
| 2.5 mg (single-dose) | 2 | 0 | 0 | 0 | 0 | 2 |
| 5 mg (single-dose) | 6 | 0 | 0 | 0 | 0 | 6 |
| 10 mg (single-dose) | 10 | 0 | 0 | 0 | 0 | 10 |
| 20 mg (single-dose) | 10 | 0 | 0 | 0 | 0 | 10 |
| 40 mg (single-dose) | 10 | 0 | 0 | 0 | 0 | 10 |
| 80 mg (single-dose) | 10 | 0 | 0 | 0 | 0 | 10 |
| 120 mg (single-dose) | 6 | 0 | 0 | 0 | 0 | 6 |
| 20 mg BID | 0 | 0 | 10 | 0 | 0 | 10 |
| 20 mg QD | 0 | 0 | 10 | 0 | 222 | 232 |
| 40 mg QD | 0 | 0 | 10 | 0 | 0 | 10 |
| 60 mg QD | 0 | 0 | 0 | 10 | 0 | 10 |
| 80 mg QD | 0 | 0 | 0 | 10 | 0 | 10 |
| 100 mg QD | 0 | 0 | 0 | 10 | 0 | 10 |
| Fast 40 mg | 0 | 14 | 0 | 0 | 0 | 14 |
| Fed 40 mg | 0 | 14 | 0 | 0 | 0 | 14 |

Table S2 Descriptive statistical results of continuous covariates

| Covariates (unit) | Median | Minimum | Maximum |
| --- | --- | --- | --- |
| Age (year) | 33.0 | 18.0 | 69.0 |
| Height (cm) | 166 | 145 | 186 |
| Body weight (kg) | 61.5 | 45.0 | 86.0 |
| Body surface area (m^2^) | 1.70 | 1.30 | 2.10 |
| BMI (kg/m^2^) | 22.7 | 18.0 | 30.9 |
| ALT (U/L) | 14.9 | 4.80 | 70.4 |
| AST (U/L) | 20.0 | 10.2 | 53.0 |
| Total protein (g/L) | 73.1 | 63.3 | 94.7 |
| T-Bil (μmol/L) | 12.0 | 4.60 | 43.8 |
| Direct bilirubin (μmol/L) | 3.60 | 1.30 | 14.9 |
| ALP (U/L) | 65.0 | 26.0 | 234 |
| Creatinine (μmol/L) | 70.0 | 44.3 | 104 |
| WBC (*10^^9^/L) | 5.86 | 3.20 | 11.0 |
| NEU (%) | 61.1 | 38.8 | 83.2 |
| LYM (%) | 30.2 | 13.0 | 49.6 |
| RBC (*10^^12^/L) | 4.73 | 3.27 | 7.55 |
| Hematocrystallin (g/L) | 140 | 88.0 | 178 |
| BPC (*10^^9^/L) | 233 | 119 | 554 |
| Creatine kinase (U/L) | 94.0 | 41.2 | 435 |
| APTT (s) | 33.2 | 19.1 | 42.4 |
| PT (s) | 11.1 | 9.20 | 14.1 |
| LBM (kg) | 46.9 | 37.7 | 56.9 |
| CrCl (mL/min) | 107 | 64.9 | 160 |
| GFR (mL/min/1.73 m^2^) | 96.3 | 56.2 | 152 |

BMI: Body Mass Index; ALT: alanine aminotransferase; AST: aspartate aminotransferase; T-Bil: total bilirubin; ALP: alkaline phosphatase; WBC: white blood cell count; NEU: neutrophilic granulocyte percentage; LYM: Lymphocyte percentage; RBC: red blood cell count; BPC: [blood platelet count](javascript:;); APTT: activated partial thromboplastin time; PT: prothrombin time; LBM: lean body mass; CrCl: creatinine clearance rate; GFR: glomerular filtration rate

Table S3 Descriptive statistical results of categorical covariates

| Covariates | category | Count | Percentage (%) |
| --- | --- | --- | --- |
| Gender | Male/female | 120/89 | 57.4/42.6 |
| Urine protein | -/+ | 188/21 | 90.0/10.0 |
| Acetone body | -/+ | 184/25 | 88.0/12.0 |
| Race | Chinese | 209 | 100 |
| Nation | Han/Others | 202/7 | 96.7/3.34 |
| Duodenal ulcer? | No/Yes | 126/83 | 60.3/39.7 |
| rs2242480 | Wild/heterozygous/homozygous | 48/32/3 | 57.8/38.6/3.61 |
| rs776746 | Wild/heterozygous/homozygous | 4/51/28 | 4.81/61.4/33.7 |
| rs12769205 | Wild/heterozygous/homozygous | 39/35/9 | 47.0/42.2/10.8 |
| rs3758581 | Wild/heterozygous/homozygous | 0/24/59 | 0/28.9/71.1 |
| rs4986893 | Wild/homozygous | 70/13 | 84.3/15.7 |
| Diprivan | With/without | 18/65 | 21.7/78.3 |
| Dimeticone | With/without | 45/38 | 54.2/45.8 |
| Dyclonine | With/without | 31/52 | 37.3/62.7 |
| Pronase | With/without | 51/32 | 61.4/38.6 |

Table S4. Value ranges of PK parameters of parent drug and the metabolite M21-1 according to quartile or binary principle

| Category | Parameter | P25 | P50 | P75 | (min, max) |
| --- | --- | --- | --- | --- | --- |
| Anaprazole | C_max_ (N=223) | 333.55 | 504.00 | 880.00 | (77.70, 7550.00) |
|  | AUC_0-t_ (N=223) | 1378.75 | 1847.08 | 2644.57 | (228.00, 16600.00) |
|  | C_ss_min_ (N=114) | 0.00 | 3.07 | 8.39 | (0.00, 31.69) |
|  | AUC_ss_ (N=143) | 1646.97 | 2125.71 | 3127.36 | (754.00, 70989.05) |
| Metabolite M21-1 | C_max_ (N=113) | 17.57 | 37.15 | 53.96 | (17.57, 432.00) |
|  | AUC_0-t_ (N=113) | 175.45 | 422.47 | 570.58 | (175.45, 6450.05) |
|  | C_ss_min_ (N=113) | 0.70 | 5.42 | 8.29 | (0.70, 80.80) |
|  | AUC_ss_ (N=113) | 193.35 | 502.04 | 682.12 | (193.35, 5632.70) |

Table S5. Single factor analysis (logistic regression) of influencing factors to mild gastrointestinal system diseases

| Dependent variable | Independent variable | P value | Odds Ratio (95% CI) |
| --- | --- | --- | --- |
| Mild gastrointestinal system diseases (occurred/not occurred) | Parent C_max_ | 0.1437 | 1.000 (1.000, 1.001) |
|  | Parent AUC_ss_ | 0.0667 | 1.000 (1.000, 1.000) |
|  | M21-1 AUC_0-t_ | 0.1447 | 1.000 (1.000, 1.001) |
|  | M21-1 AUC_ss_ | 0.0208 | 1.000 (1.000, 1.001) |

Table S6. Single factor analysis (logistic regression) of influencing factors to various examinations

| Dependent variable | Independent variable | P value | Odds Ratio (95% CI) |
| --- | --- | --- | --- |
| Various examinations (occurred/not occurred) | Parent AUC_ss_ | 0.0042 | 1.000 (1.000, 1.001) |
|  | M21-1 AUC_0-t_ | 0.0435 | 1.001 (1.000, 1.002) |
|  | M21-1 AUC_ss_ | 0.1026 | 1.000 (1.000, 1.001) |


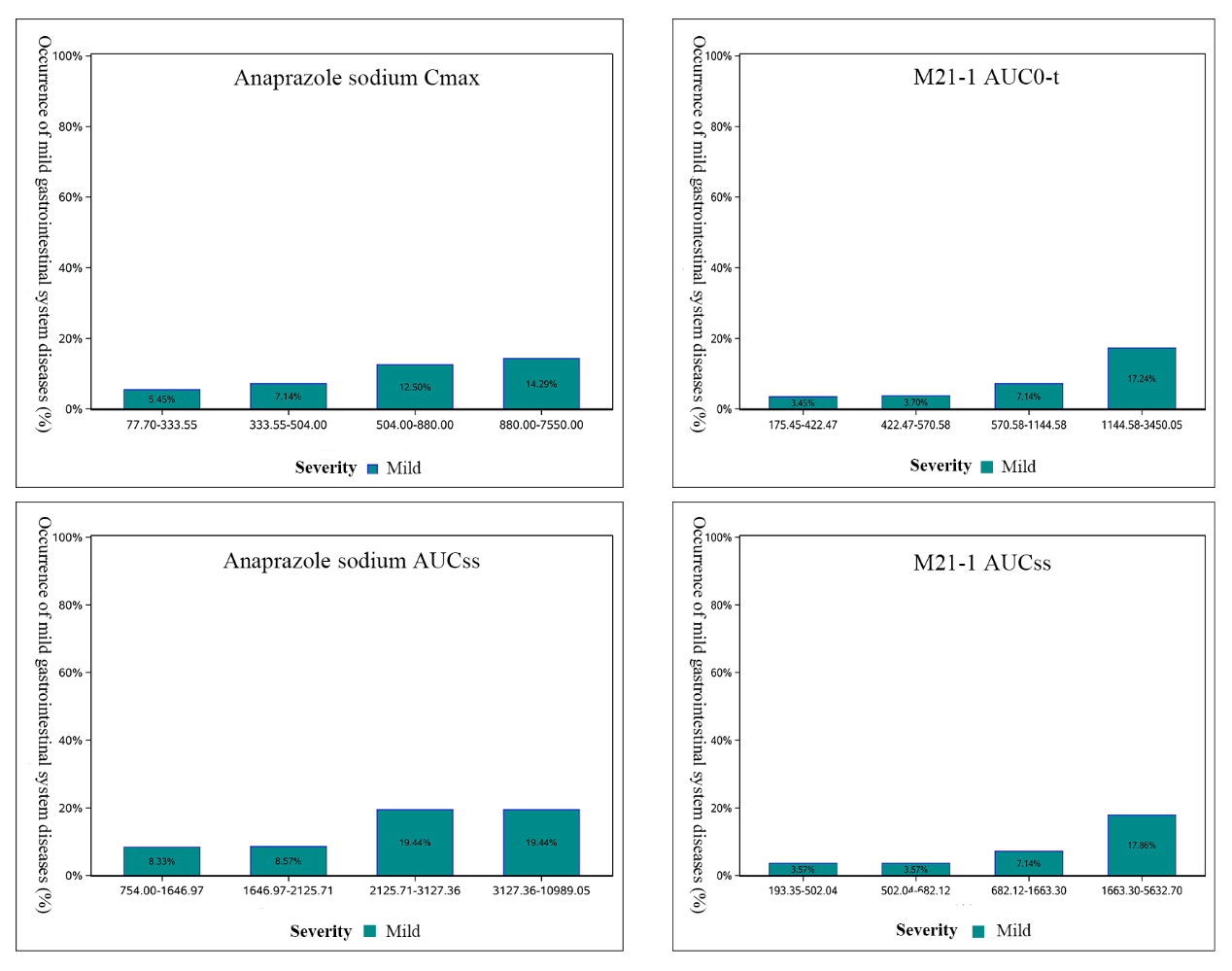


Figure S1. Occurrence of mild gastrointestinal system diseases among different C_max_ or AUC ranges of Anaprazole sodium and its metabolite M21-1


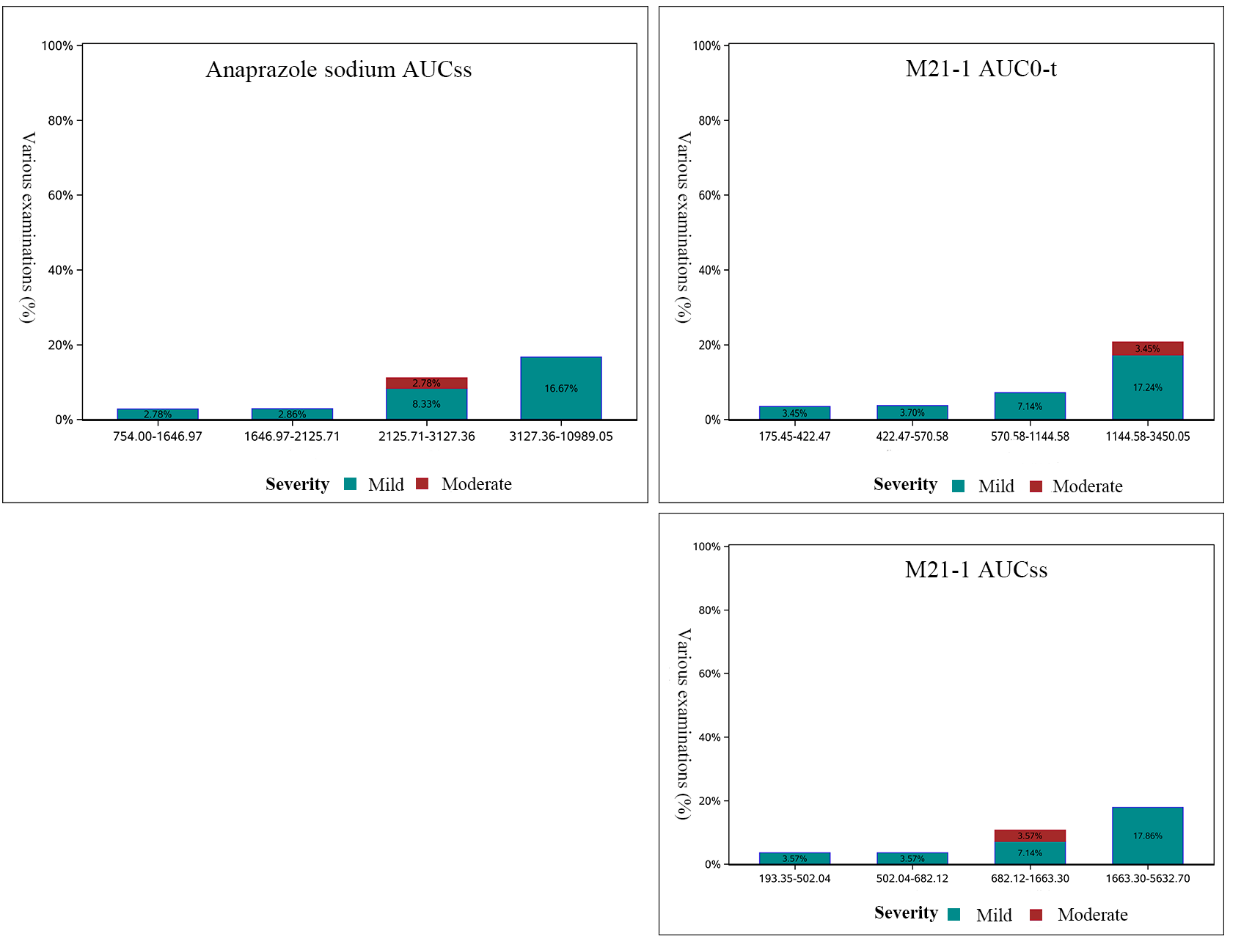


Figure S2. Occurrence of various examinations among different AUC ranges of Anaprazole sodium and its metabolite M21-1
